# Investigating Effects of Fused-Deposition Modeling (FDM) Processing Parameters on Flexural Properties of ULTEM 9085 using Designed Experiment

**DOI:** 10.3390/ma11040500

**Published:** 2018-03-27

**Authors:** Aboma Wagari Gebisa, Hirpa G. Lemu

**Affiliations:** Department of Mechanical and Structural Engineering and Materials Science, University of Stavanger, N-4036 Stavanger, Norway; aboma.w.gebisa@uis.no

**Keywords:** flexural property, full factorial design, ULTEM 9085, fused-deposition modeling, optimization

## Abstract

Fused-deposition modeling (FDM), one of the additive manufacturing (AM) technologies, is an advanced digital manufacturing technique that produces parts by heating, extruding and depositing filaments of thermoplastic polymers. The properties of FDM-produced parts apparently depend on the processing parameters. These processing parameters have conflicting advantages that need to be investigated. This article focuses on an investigation into the effect of these parameters on the flexural properties of FDM-produced parts. The investigation is carried out on high-performance ULTEM 9085 material, as this material is relatively new and has potential application in the aerospace, military and automotive industries. Five parameters: air gap, raster width, raster angle, contour number, and contour width, with a full factorial design of the experiment, are considered for the investigation. From the investigation, it is revealed that raster angle and raster width have the greatest effect on the flexural properties of the material. The optimal levels of the process parameters achieved are: air gap of 0.000 mm, raster width of 0.7814 mm, raster angle of 0°, contour number of 5, and contour width of 0.7814 mm, leading to a flexural strength of 127 MPa, a flexural modulus of 2400 MPa, and 0.081 flexural strain.

## 1. Introduction

**Figure 1:**
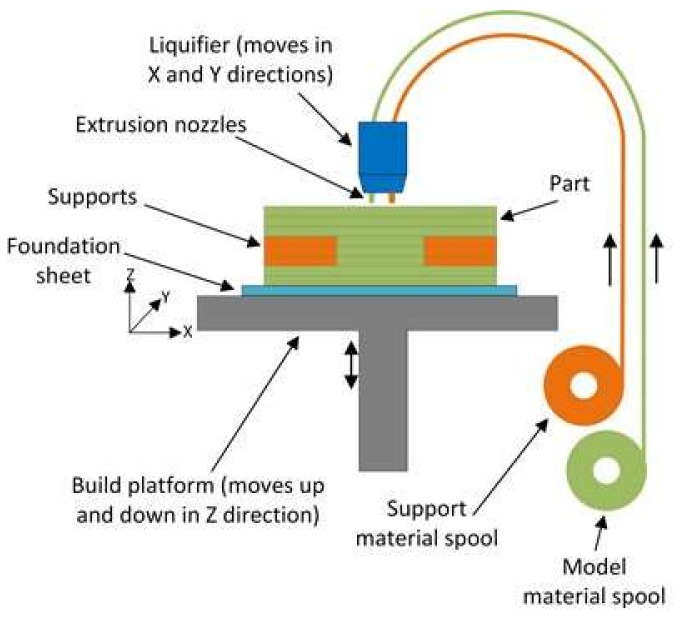
Schematic of fused-deposition modelling (FDM) process.

Additive manufacturing (AM) is an emerging manufacturing technique that has opened up new opportunities for producing complex functional structures without the need for any tooling. In the ASTM standard, AM is defined as “a process of joining materials to make objects from 3D-model data, usually layer upon layer, as opposed to subtractive manufacturing methodologies. Synonyms: additive fabrication, additive processes, additive techniques, additive layer manufacturing, layer manufacturing, and freeform fabrication” [1]. AM has several advantages over traditional manufacturing, some of which are: printing fully-functional systems, mass customization, decentralized manufacturing, and on-demand manufacturing [2]. Fused-deposition modeling (FDM), shown schematically in Figure 1, is among the categories of AM specifically defined as “a material extrusion process used to make thermoplastic parts through heated extrusion and deposition of materials layer by layer” [1]. FDM produces polymeric/plastic parts from polymeric filament by melting material, then extruding and depositing it by adjusting the different processing parameters of the building machines.

A review of the relevant literature (Section 2) shows that the technology has undergone many advancements in the last two decades. However, the choice of the right combination of process parameters still needs further investigation. Moreover, optimal process parameters obtained for one material may not work well for another material, due to differences in the material inter-filament bonding and the build temperature for different materials. Studies that have focused on flexural properties of ULTEM 9085 material are limited in number. Moreover, these studies have investigated the effect of only a few FDM process parameters. The material investigated in this study is a high-performance polymeric material that has potential applications in the aerospace, automotive and military because of its high strength-to-weight ratio and its FST (flame, smoke and toxicity) rating. In order for the industries to use this material, the mechanical properties of the material and the effect of each of the process parameters on the performance of the material need to be investigated. To fill this gap, the current study investigates the effect of five FDM process parameters on the flexural properties of the material using an experiment with a full factorial design.

Compared with previous studies on the same topic (Section 2), the work reported in this paper differs in following ways;

✓ The current study makes use of the full factorial design of an experiment in which all possible combinations of the factors are investigated instead of using other types of design that miss some of the factors combinations.
✓ This study investigates the flexural property of the ULTEM 9085 material that is relatively new and has not been explored enough, unlike most of the existing literature that has focused on tensile and compressive properties of few known thermoplastics such as acrylonitrile butadiene styrene (ABS), polycarbonate (PC) and polylactic acid (PLA).
✓ This study considered five FDM processing parameters unlike most other literatures that considered only few parameters. Moreover, this study considered one new parameter, contour width, which (to the authors’ knowledge) has not been considered in almost all of the existing literature.

The objective of the current study is to investigate the effect of five process parameters (air gap, raster width, raster angle, contour number and contour width) on the flexural property of ULTEM 9085 produced by the FDM technique. A full factorial designed experiment was employed for the investigation of the effect of individual parameters and the effect of two parameters’ interaction on the flexural property of the material. The paper is structured as follows: Section 2 discusses the review of previous works in this area. In Section 3, the materials and methods used in the study are discussed, considering the materials and AM machine, process parameters and sample preparation, design of experiment, and experimental procedure and setup. Section 4 discusses the results of the study, followed by Section 5 which draws conclusions.

## 2. Review of Previous Work

Depending on the requirements of a part’s intended application, adjustments can be made to the processing parameters in order to obtain a part that can fulfil the requirements. However, the adjustments of parameters differ from machine to machine, and the parameters have conflicting advantages. The literature plainly points out that the properties of FDM-produced parts are significantly influenced by the process parameters. Among the reported works, Chacón et al. [3] investigated the influence of process parameters on mechanical properties of additive manufactured PLA structures made by FDM. They focused on property variations with sample orientation. Among other things, they observed that ductility generally decreases with increasing feed rate and layer thickness. Mohamed et al. [4] also investigated the influence of process parameters on the dynamic mechanical performance of PC-ABS parts produced by the FDM technique. In their study, among other processing parameters, layer thickness, air gap and number of contours showed the greatest influence.

Ahn et al. [5] studied the influence of air gap, road/raster width, model temperature, material color and raster orientation on the tensile and compressive properties of ABS materials produced by the FDM technique. Their investigation revealed that the mechanical properties of FDM-produced parts are apparently parameter-dependent and anisotropic (show superior properties in the filaments’ deposition direction). They also pointed out that air gap and raster orientation have considerable influence on the mechanical properties of the material. Wu et al. [6] investigated the influence of layer thicknesses (200, 300 and 400 μm) and raster angles (0°, 30° and 45°) on the mechanical properties of 3D-printed poly ether ether ketone (PEEK) material and concluded that both factors have a similar effect on the tensile, compressive and flexural properties. Deng et al. [7] carried out a study on the optimization of the mechanical properties of PEEK material in terms of process parameters. The study suggested that the optimal mechanical properties can be obtained for the process combinations of a printing speed of 60 mm/s, layer thickness of 0.2 mm, temperature of 370 °C and filling ratio of 40%. Casavola et al. [8] studied the effect of raster angle (±30°, ±45°, 0°/90° and 0° only) on the residual stresses that develop due to the rapid heating and cooling nature of the process in FDM-produced parts of ABS material. Their study concluded that the residual stress is higher, i.e., is the worst configuration, for parts built with ±30° raster angle, whereas the part with the lowest residual stress, i.e., the best configuration, is the one built with ±45°.

Onwubolu and Rayegani [9] carried out a study on the influence of layer thickness, part orientation, raster angle, raster width, and air gap on the tensile property of ABS parts produced by FDM technology. Their investigation indicated that the optimum combination of parameters that could improve the tensile properties of the parts is minimum layer thickness, negative air gap, minimum raster width, and increased raster angle. Bagsik et al. [10] studied the effect of build direction, with a default raster orientation, on the tensile and compressive properties of ULTEM 9085 material produced by the FDM technique. From their investigation, they concluded that the edge build direction gives higher tensile strength, compared to the flat and upright build directions. Their study also showed that the highest compressive strength is registered for the part built in the upright (Z) direction, rather than in the other two build directions. Bagsik and Schöppner [11] also extended their investigation, considering more parameters such as raster angle (0°, 30° and 45°), build orientation (flat, edge and upright), raster width (thin and thick) and raster-to-raster and raster-to-perimeter air gap (negative and positive). Their investigation was to discover the influence of these parameters on the tensile property of the same material produced by the same FDM technique. Their study showed that the highest tensile strength was achieved for all build directions using a negative raster air gap. Their investigation also disclosed that the improvement of the tensile properties could be achieved by using thick filaments for both edge and upright build directions. However, the tensile property for parts built in flat build orientation could be improved by employing a thinner filament. Motaparti et al. [12] also carried out a study on the effect of build parameters on the compression property of ULTEM 9085 produced by the FDM technique. In their investigation, they considered three parameters (build direction, raster angle, and air gap) and two types of specimen (solid and sparse) with a full factorial design experiment. They concluded that the interaction between two parameters, build direction and raster angle, significantly affects the compressive yield strength of the material.

Christiyan et al. [13] also studied the influence of process parameters (layer thickness and printing speed) on the mechanical properties of 3D-printed ABS composite. Based on their investigation, they outlined that low printing speed and low layer thickness resulted in the highest tensile and flexural strength of the material. Motaparti et al. [14] also investigated the effect of parameters, build direction, raster angle and negative air gap on the flexural properties of ULTEM 9085 produced by the FDM technique with solid- and sparse-build styles. Their investigation revealed that the vertical (edge) build direction could result in higher flexural yield strength than its horizontal counterpart.

## 3. Materials and Methods

### 3.1. Material and Additive Manufacturing (AM) Machine

The material used in this investigation is Polyetherimide (ULTEM 9085) filament—known for its high flame retardance—a high-performance thermoplastic polymer [15], supplied by Stratasys. Due to the material’s high strength-to-weight ratio and its FST (flame, smoke and toxicity) rating, it is considered ideal for the transportation industry (automotive and aerospace) and for the military. The machine employed for preparation of the samples is Fortus 450 mc [16], supplied by Stratasys. The machine has a large build envelope of 406 × 355 × 406 mm^3^.

### 3.2. Process Parameters and Sample Preparation

**Figure 2:**
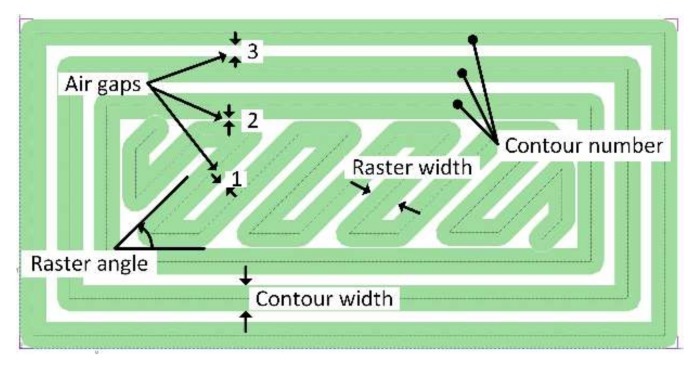
FDM process parameters.

As discussed in Section 1, the mechanical properties of FDM-produced parts are apparently dependent on the processing parameters. The process parameters shown in Figure 2 are selected for this study based on the literature [7,9,17] and preliminary study in the field. All considered parameters are controllable by the user and are detailed as follows:

- Air gaps: these are the distances or spaces between two adjacent rasters, as represented by number 1 in Figure 2, or between a raster and a contour, as represented by number 2 in Figure 2, or between two adjacent contours, as represented by number 3 in Figure 2.
- Raster width: this is the width of the material bead used for rasters.
- Raster angle: this is the angle measured from the X-axis (horizontal axis) of a layer to a raster.
- Contour number: this is the number of closed toolpaths that follow the outline of a region of a part.
- Contour width: this is the width of the material bead used for contours. This parameter was not included in the previous studies reported in the introduction section. This could be due to the assumption that the road width for raster and contour can have a similar effect on the properties of the materials.

After the process parameters that are applicable to the material and the AM machine considered were selected, and 32 samples with two replicates, 64 samples, with different parameters combinations were prepared for the investigation. Except for the selected parameters, all the other parameters of the AM machine were set to the default values. Before the production of the samples, the parameter combinations were performed with a custom group using INSIGHT 11.5 software (Stratasys, Eden Prairie, MN, USA) supplied with the AM machine.

### 3.3. Design of Experiment

For the investigation into the effect of process parameters on flexural properties of the material, a full factorial design for the experiment was employed, since the primary purpose of the investigation is to screen out the important effects from the less important ones. A commercially available software, Design Expert version 10.0.8 (Statease, Minneapoli, MN, USA), was employed for this purpose. In the investigation, five process parameters with two levels, low and high, detailed in Table 1, were considered. Preliminary tests were conducted to select the appropriate values for the levels of the parameters. As air gap is considered as a defect in FDM parts, it should be eliminated as much as possible to obtain dense structures. One way to do this is by adjusting the air gap parameter to negative values that is available in the FDM machines. However, the adjustment of the air gap to negative values brings other problems, such as poor surface quality and poor dimensional tolerances [18], because of extra material building up during deposition. On the other hand, the extra material buildup and surface roughness problem can be solved by making adjustments (slowing) on the extrusion rate. However, with the FDM machine (Fortus 450 mc) used in this investigation, the adjustment of the extrusion or deposition rate is not possible. Thus, the only solution is to find optimal process parameter combinations that can result in a dense structure to improve the performance of the components excluding the very negative air gap. As a result, the low and high levels for the air gap were selected as −0.0254 mm and 0.000 mm, respectively.

The low and high levels for raster width and contour width were selected to be 0.4064 mm and 0.7814 mm as minimum and maximum values, respectively; these are available in the Fortus 450 mc FDM machine. The contour number levels were selected to fit the size of the specimen dimension as one and five for low and high, respectively. The selected two levels of raster angle were the two extreme values of interest to study their effects.

With the full factorial design of the experiment and five factors (processing parameters), 2^k^ runs i.e., 2^5^ = 32 runs in two replicates, 64 in total, were performed. Table 2 shows the design matrix with the average experimental results obtained from two replications of the flexural test. The low and high levels of the factors are coded into −1 and +1, respectively, using Equations (1) and (2) [19]. In this investigation, a regression model with two-factor interaction (2FI) is proposed with the assumption that the two factor interactions simplify the analysis. For prediction of the responses, *Y* is given by Equation (3).

(1) $$X_{low\;coded\;}\left(-\right)=\frac{A_{low}-\left(A_{low}+A_{high}\right)/2}{\left(A_{high}-A_{low}\right)/2}$$

(2) $$X_{high\;coded\;}\left(+\right)=\frac{A_{high}-\left(A_{low}+A_{high}\right)/2}{\left(A_{high}-A_{low}\right)/2}$$

where *A_low_* is the low-level value of the factors, and *A_high_* is the high-level value for the factors.

(3) $$Y=\beta_{0\;}+\overset{5}{\underset{i=1}{\sum }}\beta_{i}X_{i}+\overset{5}{\underset{i<j}{\sum }}\beta_{ij}X_{i}X_{j}+\varepsilon$$

where *β*s are the regression coefficients, *X* represent the coded factors (parameters), and *ε* is the random error.

For the current investigation, which considered five parameters represented by symbols *A*, *B*, *C*, *D* and *E* in Table 1, the regression model can be rewritten as given by Equation (4):

(4) $$Y=\beta_{0\;}+\beta_{1\;}A+\beta_{2\;}B+\beta_{3\;}C+\beta_{4\;}D+\beta_{5\;}E+\beta_{12\;}AB+\beta_{13\;}AC+\beta_{14\;}AD+\beta_{15\;}AE+\;\beta_{23\;}BC+\beta_{24\;}BD+\beta_{25\;}BE+\beta_{34\;}CD+\beta_{35\;}CE+\beta_{45\;}DE+\varepsilon$$

where *Y* is the predicted response, *β*s are the regression coefficients, *A*, *B*, *C*, *D* and *E* represent the main effects of the independent coded variables or factors, combinations like *AB*, *AC* and so on are the two-factor interaction effects of the individual factors, and *ε* is the random error.

### 3.4. Experimental Procedures and Setup

**Figure 3:**
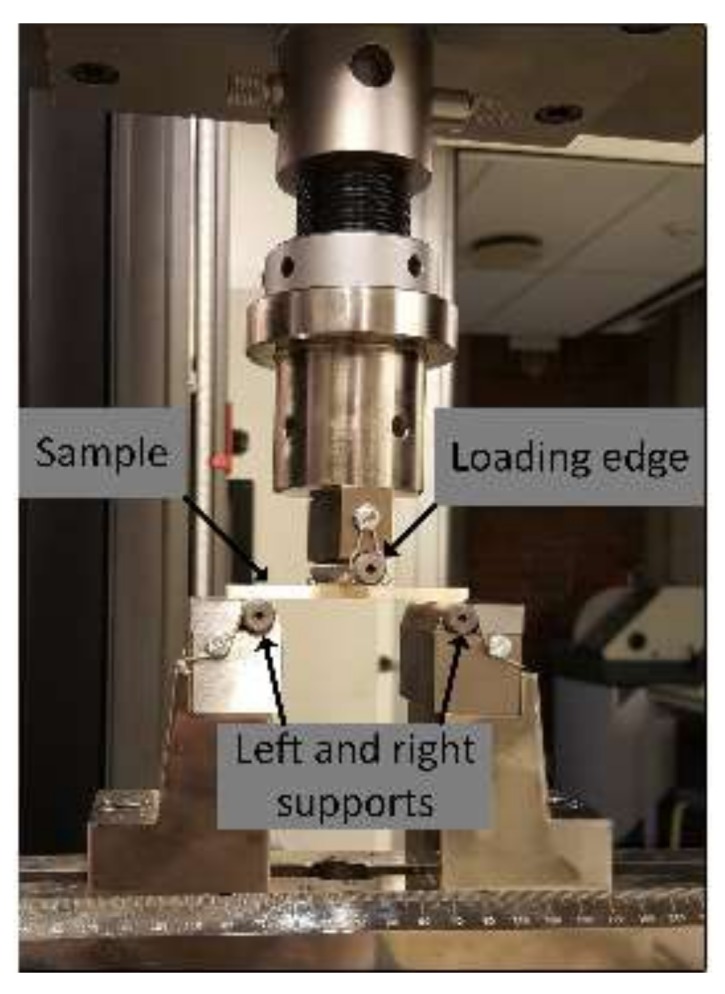
Experimental setup for flexural test.

For the current experimental investigation, the INSTRON 5985 universal testing machine with a load cell of 250 kN was used. The experiment was conducted by adopting ISO 178 standard [20], based on recommendations from the standard organizations, International Standards Organization (ISO) [21] and National Institute of Standards and Technology (NIST) [22], since no specific standard has been developed for the testing of parts produced by the FDM technique. The flexural test was carried out by placing the specimen on two supports from the bottom and applying the load from above with the loading edge of radius 5 mm, as shown in the experimental setup in Figure 3. A recommended speed of 2 mm/min was used for the test as per the standard. A 10 N preload was applied before the test started to ensure good contact between the specimen and the supports. Once the load reached the maximum, for the samples that break, the test was stopped. However, for the samples that did not break at the maximum load, 5% strain was used as the stopping criterion, as per the recommendation in the standard.

## 4. Results and Discussion

**Figure 4:**
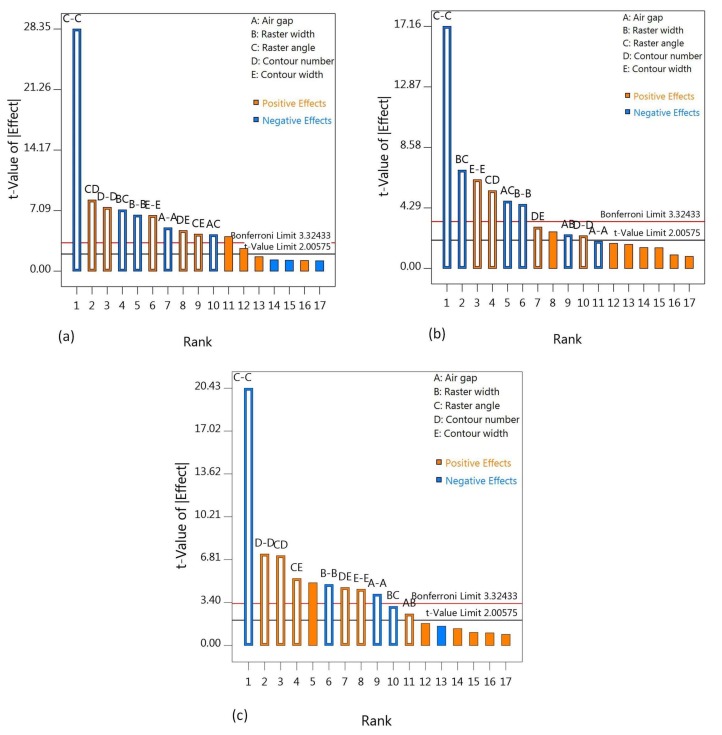
Factor screening using Pareto charts for (**a**) flexural strength; (**b**) modulus and (**c**) strain.

Design-Expert version 10.0.8 software was employed for the analysis of the data from the experiment. Based on the discussion in Section 3.3, regression models have been developed after significant and insignificant factors have been identified using Pareto charts, as shown in Figure 4. In the Pareto charts, combinations of similar characters, such as A-A, represent the single-factor effects, whereas the dissimilar-character combinations, such as BC, represent the two-factor interaction effects. For the screening of the effects, Pareto uses two different t limits, the Bonferroni or family-wise corrected t-critical value and the standard t-critical. Moreover, the yellow color shows that the factors have a positive effect on the response, whereas the blue color represents the negative effect of the factors on the response. Regression models in Equations (5)–(7) are developed considering only the significant factors shown in the Pareto charts. The positive and negative signs in the regression models show the synergetic and antagonistic effects of the factors, respectively.

(5) $${\left(\mathrm{Flexural}\;\mathrm{strength}\right)}^{2.75}=4.186\;x\;10^{5}-28,522.8A-36,931.71B-1.595\;\times \;10^{5}C+42,019.9D+36,665.02E-23,994.42\mathrm{AC}-40,355.09\mathrm{BC}+46,839.07\mathrm{CD}+24,343.63\mathrm{CE}+26,693.89\mathrm{DE}$$

(6) $${\left(\mathrm{Flexural}\;\mathrm{modulus}\right)}^{3}\;=1.076\;\times \;10^{10}-\;2.259\;\times \;10^{8}A\;-\;5.339\;\times \;10^{8}B-2.018\;\times \;10^{9}C+2.722\;\times \;10^{8}D+7.389\;\times \;10^{8}E-2.804\;\times \;10^{8}\mathrm{AB}-5.61\;\times \;10^{8}\mathrm{AC}-8.192\;\times \;10^{8}\mathrm{BC}+6.474\;\times \;10^{8}\mathrm{CD}+3.442\;\times \;10^{8}\mathrm{DE}$$

(7) $${\left(\mathrm{Flexural}\;\mathrm{Strain}\right)}^{2.77}=6.797\times \;10^{-4}-5.443\;\times \;10^{-5}A-6.471\;\times \;10^{-5}B-2.737\;\times \;10^{-4}C+9.714\;\times \;10^{-5}D+5.966\;\times \;10^{-5}E+3.34\;\times \;10^{-5}\mathrm{AB}-\;4.141\;\times \;10^{-5}\mathrm{BC}+9.537\times \;10^{-5\;}\mathrm{CD}+\;7.1\;\times \;10^{-5}\mathrm{CE}+\;6.15\;\times \;10^{-5}\mathrm{DE}$$

**Figure 5:**
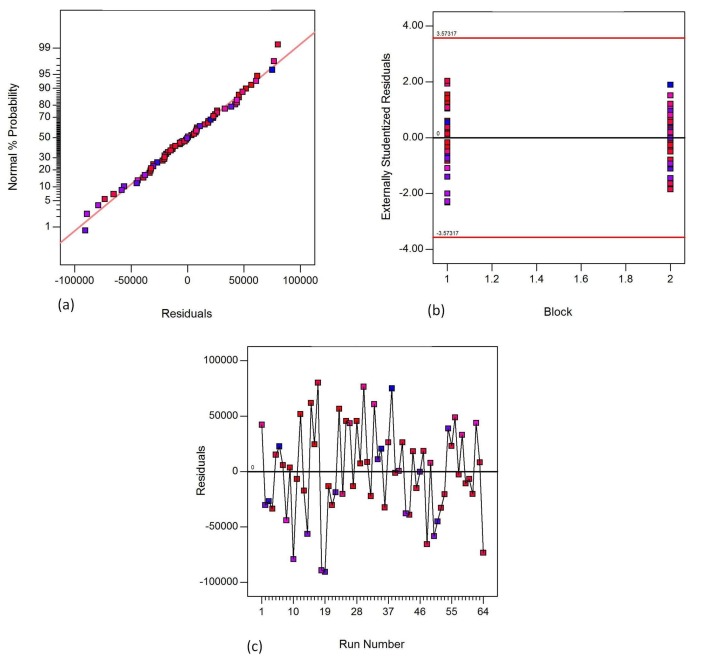
Fisher assumptions (**a**) residual normality; (**b**) equality of variance and (**c**) run-order independence for flexural strength.

**Figure 6:**
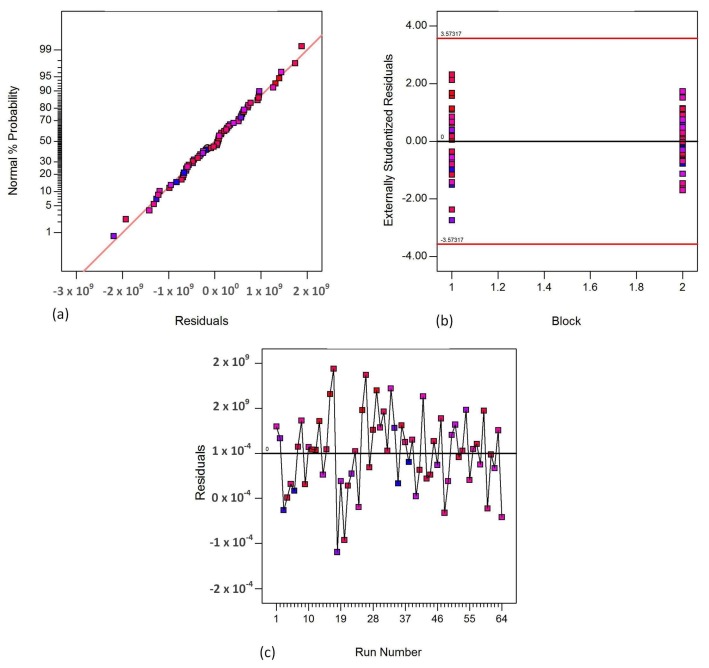
Fisher assumptions (**a**) residual normality; (**b**) equality of variance and (**c**) run-order independence for flexural modulus.

**Figure 7:**
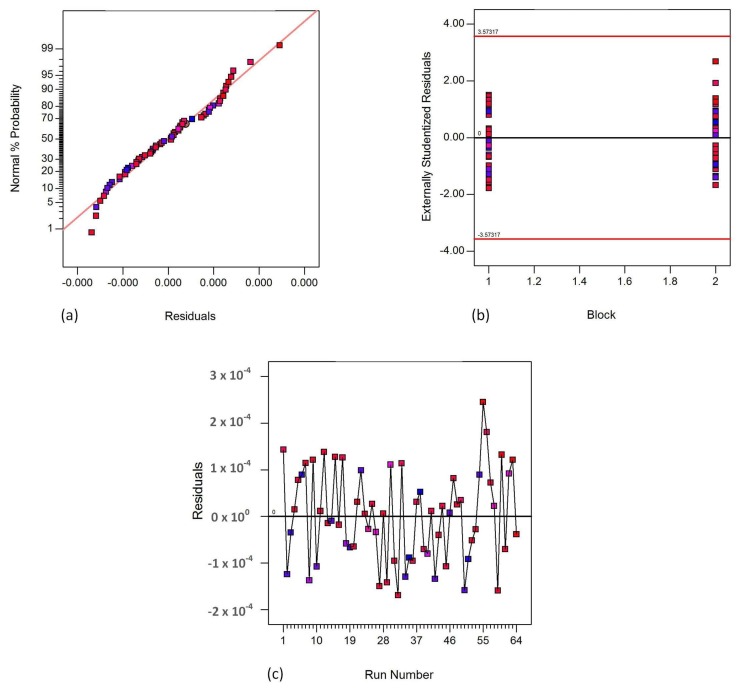
Fisher assumptions (**a**) residual normality; (**b**) equality of variance and (**c**) run-order independence for flexural strain.

Figure 5, Figure 6 and Figure 7 show the fisher assumptions test for the models developed. The linear patterns in Figure 5a, Figure 6a and Figure 7a show the linearity of the residuals in both replicates for all the responses, flexural strength, flexural modulus and flexural strain. The linearity of the patterns in the plots are evident for the good-fitting of the predicted values to the experimental (actual) values. The plots in Figure 5b, Figure 6b and Figure 7b show the equality of variance for both replicates (Block 1 and Block 2). These plots demonstrate that the runs in Block 1 and in Block 2 have a similar variation of residuals. Figure 5c, Figure 6c and Figure 7c demonstrate the run-order independence of the runs. These plots show patterns that show that the residuals in the model are randomly distributed.

Analysis of variance (ANOVA) is used to assess the test of the significance of the process parameters or factors and the interaction between the factors. In the ANOVA, factors are regarded as significant if the probability (Prob > F value) is very small, i.e., less than 0.05. Table 3 shows the details of the ANOVA analysis: the significant factors are tested with Prob > F value lower than 0.05. Furthermore, the good agreement seen between adjusted and predicted R^2^, i.e., within 0.2 of each other and an adequate precision of over four, also show the significance of the model.

Insignificant factors, AB, AD, AE, BD and BE for flexural strength; AD, AE, BD, BE and CE for flexural modulus; and AC, AD, AE, BD and BE for flexural strain with Prob > F value higher than 0.05 are excluded from the models.

### 4.1. Effect of Air Gap

**Figure 8:**
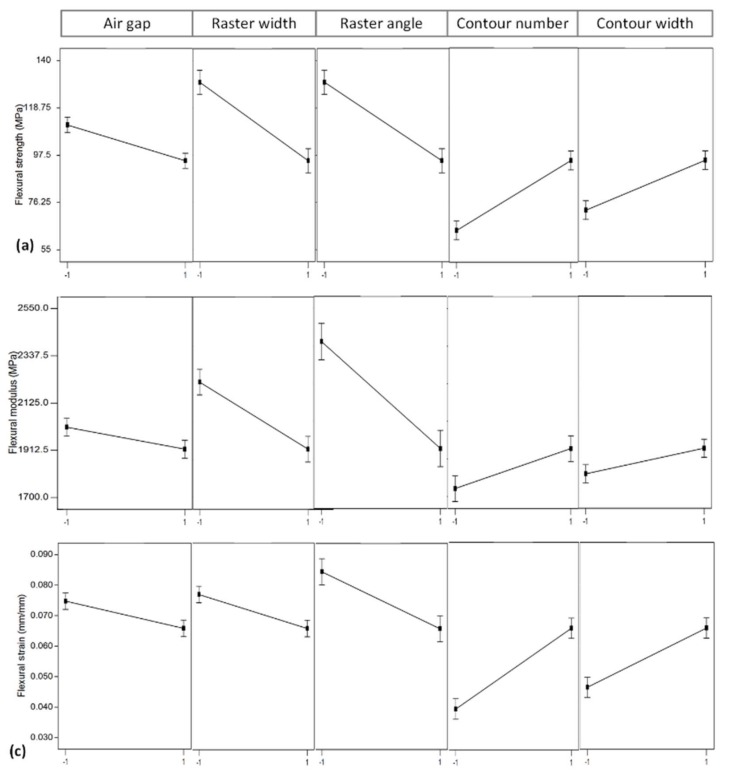
Plots showing main effects of process parameters on (**a**) flexural strength; (**b**) flexural modulus; and (**c**) flexural strain.

**Figure 9:**
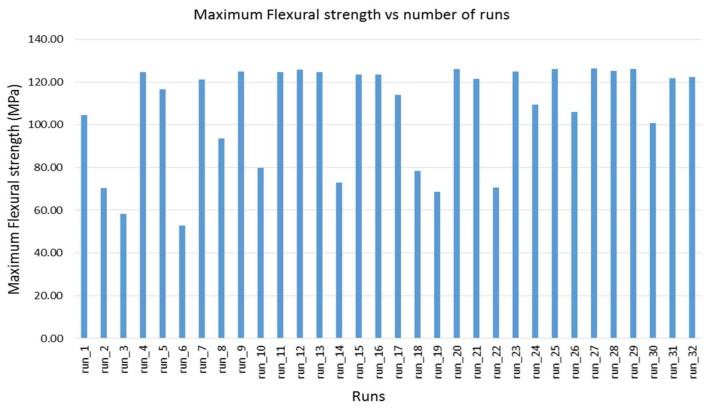
Chart showing the variation of maximum flexural strength of ULTEM 9085 resulting from the variation of the process parameters’ combination.

**Figure 10:**
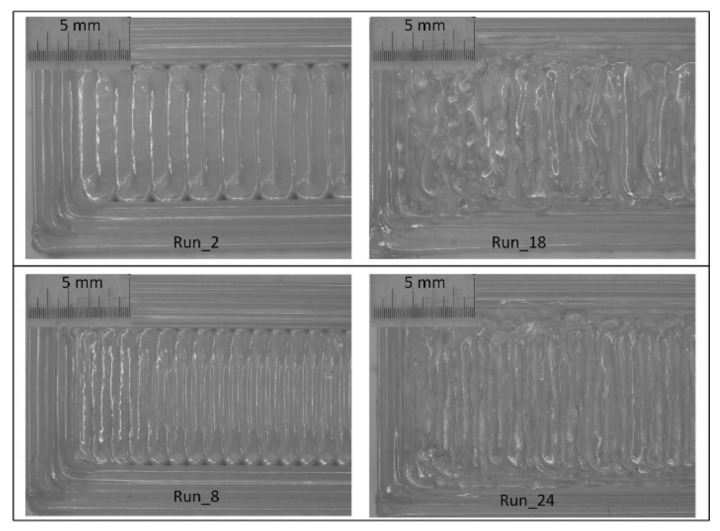
Effect of air gap on the surface quality of parts.

The effect of the air gap on the flexural property of the material is not great, compared to the other parameters, as shown in Figure 8a–c. However, it makes its own contribution to the variation of the flexural properties of the material, as shown in Figure 9, which illustrates the maximum flexural strength variations of all the runs. Among the runs, one to 16 are produced with zero air gap (0 mm), while the rest are produced with a negative air gap (−0.0254 mm). It is very clear from Figure 9 that, when all the counter runs (i.e., low and high air gaps) such as run_1 and run_17, run_2 and run_18, and so on are compared, the negative air gap has improved the flexural strength of the material. This could be due to the dense structure obtained by slightly overlapping deposition of the filaments, thus avoiding the gap between the filaments. The negative air gap also strengthens the adhesion between the adjacent filaments so that the flexural properties of the material are improved. However, the negative air gap also has drawbacks in reducing the dimensional accuracy and the surface quality of the part, as shown in Figure 10, which displays the comparison of a few representative counter parts, run_2 and run_18, and run_8 and run_24, with positive and negative air gaps, respectively, while the other parameters are unchanged. From the figure, it is clear that the surface quality of those parts with a negative air gap is very poor, compared to their counterparts with a positive air gap that resulted from the overlapping of the filaments and excess material buildup.

It is worth mentioning that there are conflicting reports on the effect of air gap on the mechanical properties of FDM-processed ABS material. Ahn et al. [8] reported that a negative air gap improves the mechanical properties of the material (ABS), but they limited the lower value of the air gap to be not lower than −0.0762 mm, as excess material can be accumulated on the nozzle and the part itself. Dawoud et al. [23] also reported that a negative air gap of 0.05 mm can improve the mechanical properties of the material, but they did not limit the value of the air gap. However, Mohammed et al. [4] and Sood et al. [24] reported, on the contrary, that lower values of air gap can result in the development of stress accumulation through the restriction of heat transfer or heat loss, since the filaments are deposited very close to each other. Mohammed et al. [4] even further claimed that a positive air gap has an advantage in facilitating the spread of semi-molten materials between the gaps, resulting in structurally stronger parts.

From the investigation and past reports, it can be said that, for structural materials like ABS and PC, a negative air gap of not less than 0.05 mm can lead to improvements in mechanical properties without significant influence on their dimensional accuracy and surface quality. However, for materials like ULTEM 9085, which are processed at high temperature, an air gap of 0 mm is sufficient and, by adjusting other parameters, as can be seen in Figure 6, for runs 4, 7, 10, 12, 13, 15 and 16, the mechanical properties can be improved to balance the pros and cons of using a negative air gap.

### 4.2. Effect of Raster Width

From Figure 8a–c, it can be seen that the change in raster width has a significant influence on the flexural properties of the material. This is distinctly visible in Figure 9, comparing counterparts with low (0.4064 mm) and high (0.7814 mm) raster width, while other parameters are not changed. For example, in run_2 and run_8, run_3 and run_10, and run_6 and run_14, the higher values of raster width improve the flexural strength of the material. This can be attributed to the fact that thicker raster width can contribute to a uniform thermal gradient in the part-deposition process so that it can result in minimum distortion. Furthermore, thicker rasters can strengthen the part, as they can resist the applied load much better than thinner rasters.

### 4.3. Effect of Raster Angle

**Figure 11:**
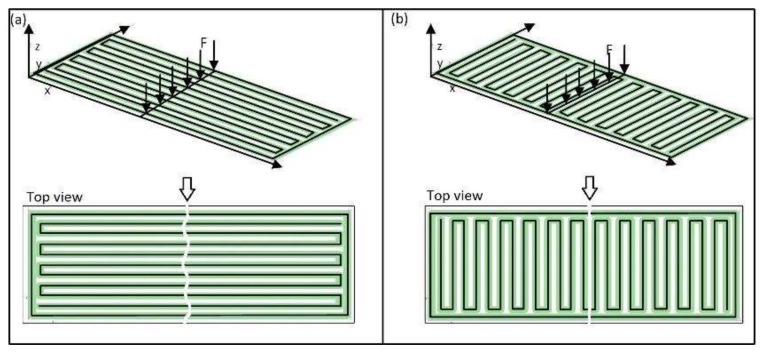
Parts manufactured with raster angle (**a**) low (0°) and (**b**) high (90°) with their loading conditions and failure modes as top view.

**Figure 12:**
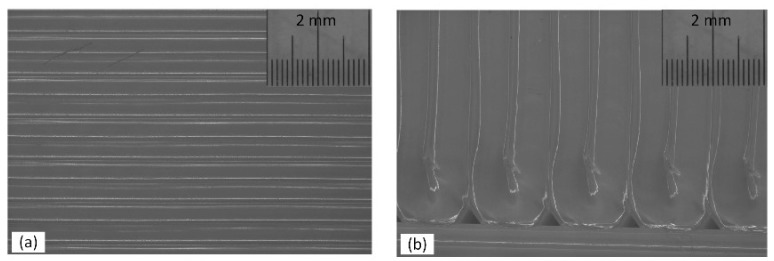
Light microscope image of: (**a**) dense structure obtained for samples built with 0° raster angle and (**b**) samples built with 90° raster angle showing incomplete fill and void at the edges.

The major effect of the raster angle on the flexural properties of the material is very clear in Figure 8a–c, where the effect of the raster angle is much greater on flexural modulus than on the other two properties. Counterparts with low (0°) and high (90°) raster angles, keeping the other parameters unchanged, were compared for instance in run_1 and run_4, run_2 and run_7, run_3 and run_9, run_10 and run_12, run_14 and run_16 and so on in Figure 6. From the figure, it is obvious that a low (0°) raster angle significantly improves the flexural properties of the material. This could be because in parts with a low (0°) raster angle, long filaments are deposited in the length of the part and the load has to cut all the filaments before the part breaks, as shown in Figure 11a. However, for parts with a high raster angle (90°), the filaments are deposited perpendicular to the filaments’ length and the flexural load cuts only a few filaments before the part breaks, as shown in Figure 8b. The other reason for the parts with a 0° raster angle (Figure 12a) performing better than parts with a 90° angle could be the dense structure obtained with filaments deposited parallel to each other in the length of the part, compared to the incomplete fill resulting for the part printed in a 90° raster angle (Figure 12b).

### 4.4. Effect of Contour Number

**Figure 13:**
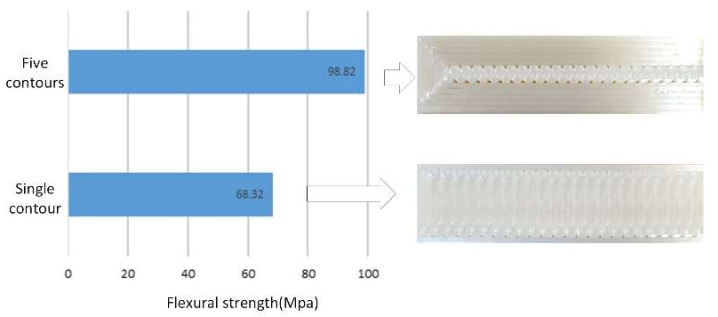
Effect of contour number on the flexural properties.

Figure 8a–c shows that the contour number has a strong effect on the flexural properties, flexural strength and flexural strain, of the material. It is clear from the plots that increasing the number of contours from low (one) to high (five) significantly increases the flexural properties of the material. This is due to the fact that increasing the number of contours results in decreasing the number of rasters and the raster length. Thus, the load applied is mainly supported by the contours rather than the rasters, which leads to improvement in the performance of the material. The effect of the number of contours is much more visible on parts produced with a 90° raster angle than on parts in the other direction. Figure 13 shows the effect of contour number on parts produced with a 90° raster angle. The values are calculated as the average of six runs each, run_1, run_2, run_5, run_8, run_17 and run_18 for parts with five contours, and run_3, run_6, run_10, run_14, run_19 and run_22 for parts with a single contour.

### 4.5. Effect of Contour Width

The effect of contour width on the flexural properties of the material is shown in Figure 8a–c. The effect can be seen in Figure 9, comparing counterparts with high (0.7814 mm) and low (0.4064 mm) contour widths, while other parameters are unchanged, such as run_1 and run_2, run_3 and run_6, run_5 and run 8, run_17 and run_18, and run_21 and run 24: the high level of contour width improves the flexural strength of the material. This could be due to the same reason as that of raster width, in that thicker contour width can contribute to a uniform thermal gradient in the part-deposition process, resulting in minimum distortion. Furthermore, a thicker contour width creates parts with thick contours that can strengthen the parts, as they can resist the applied load much better than the thinner contours. However, these effects are visible only on parts produced with a 90° raster angle. This could be attributed to the fact that parts produced with a 0° raster angle are very dense and the effect of varying contour width could not be realized. On the contrary, in parts produced with a 90° raster angle, as there are incomplete fills, the thicker contours improve the performance of the parts by lowering the number of incomplete fills or gaps.

### 4.6. Interaction Effects

**Figure 14:**
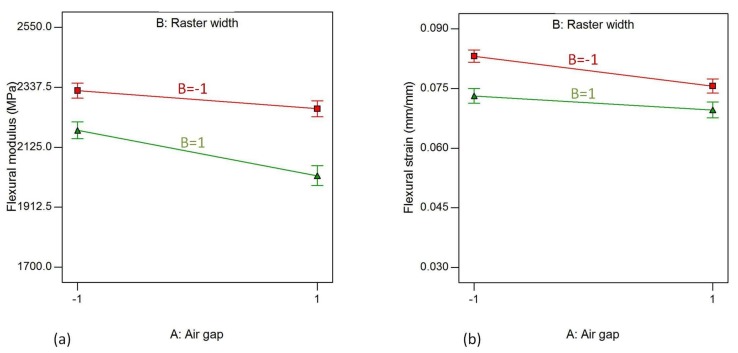
Interaction effect of air gap and raster angle on (**a**) flexural modulus and (**b**) flexural strain, while raster angle, cotour number and contour width are kept at their highest level.

In addition to the main effects of the parameters, significant effects from the interaction of parameters on the flexural properties of the material are also prominent. Figure 14a,b show the interaction effects of air gaps (A) and raster width (B) on flexural modulus and flexural strain. The AB interaction effect represents the effect of interaction of air gaps and raster width, while the other parameters are kept fixed at their high values. From the plots, it is clear that flexural modulus and flexural strain tend to decrease when the air gap increases from −0.0254 mm to 0.000 mm to for both levels of raster width with the exception of flexural strain, which shows an almost constant trend for the low level of raster width.

**Figure 15:**
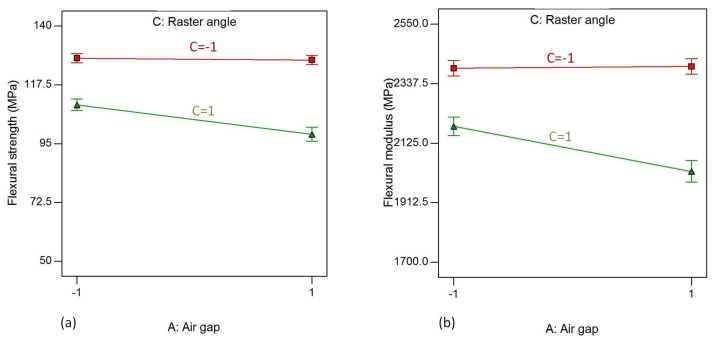
Interaction effect of air gap and raster angle on (**a**) flexural strength and (**b**) flexural modulus, while raster width, contour number and contour width are kept at their highest level.

The interaction effect of air gaps (A) and raster angle (C) on flexural strength, flexural modulus and flexural strain is shown in Figure 15. The AC interaction effect represents the effect of interaction of the air gap and raster angle, while the other parameters are kept fixed at their high values. As shown in Figure 15a (i.e., when the raster width, contour number and contour width are at their high levels), the flexural strength remain almost constant for a raster angle of 0°, while they tend to decrease with increased air gap when the raster angle is increased to 90°. On the other hand, the flexural modulus increases for a raster angle of 0° but decreases for a 90° raster angle with increasing air gap, as shown in Figure 15b.

**Figure 16:**
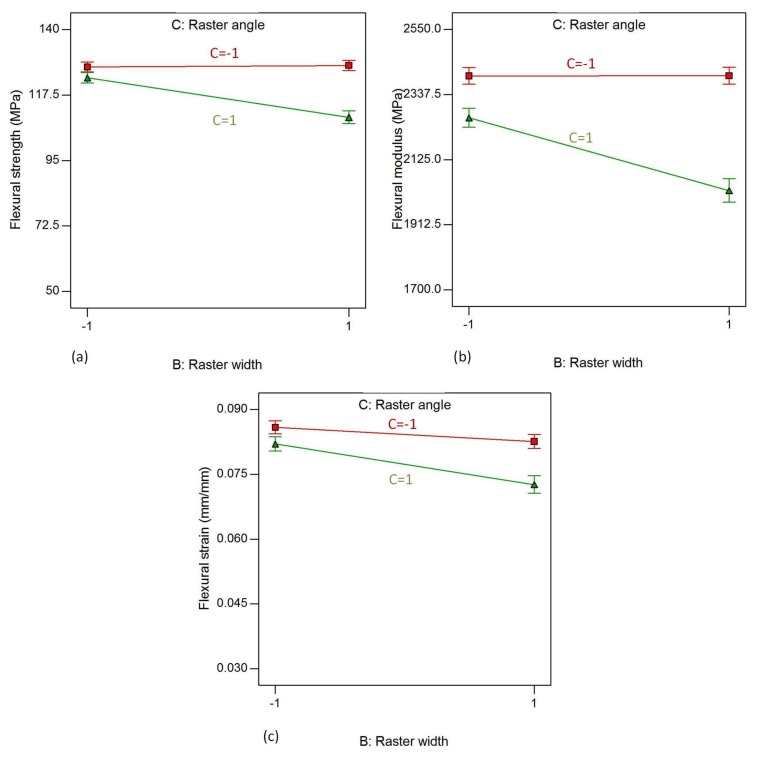
Interaction effect of raster width and raster angle on (**a**) flexural strength; (**b**) flexural modulus and (**c**) flexural strain, while air gap, contour number and contour width are kept constant at their highest level.

Figure 16 shows the interaction effects of B (raster width) and C (raster angle) on the flexural properties of the material, while raster width, contour number and contour width are kept constant at their high level. From the plots, it is clear that the trends of effects are similar for all three flexural properties. It is evident that flexural strength, flexural modulus and flexural strain exhibit an almost constant trend when the raster width increases from 0.4064 mm to 0.7814 mm for the raster angle of 0°. On the contrary, flexural strength, flexural modulus and flexural strain exhibit a decreasing trend when the raster width increases from 0.4064 mm to 0.7814 mm for the raster angle of 90°. This shows that the interaction effect is more evident on the parts with a raster angle of 90° than on those with an angle of 0°.

**Figure 17:**
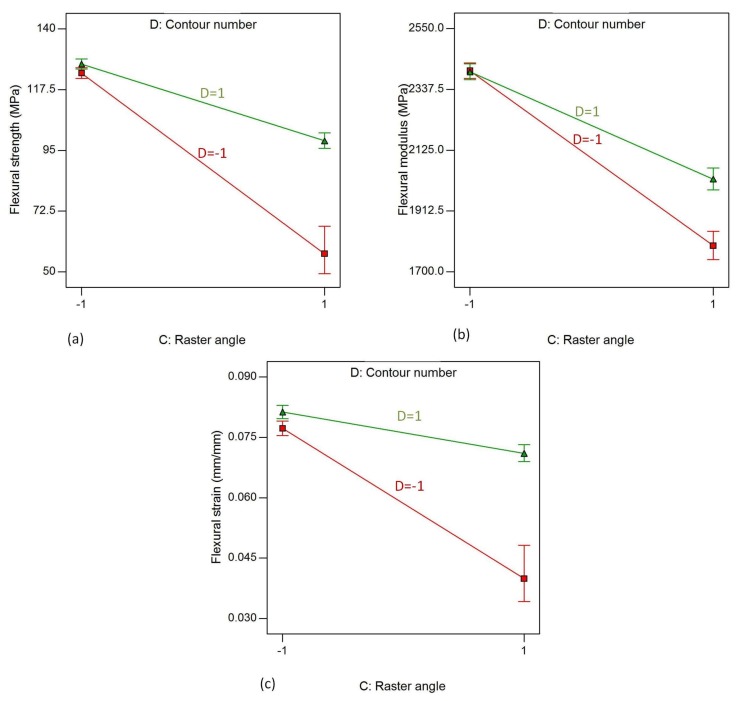
Interaction effect of raster angle and contour number on (**a**) flexural strength; (**b**) flexural modulus, and (**c**) flexural strain, while air gap, rsater width and contour width are kept at their highest level.

The interaction effects of raster angle and contour width on the responses, flexural strength, flexural modulus and flexural strain, while the other factors are kept constant at their high level, are shown in Figure 17a–c, respectively. From the figure, it is evident that flexural strength, flexural modulus and flexural strain tend to decrease when the raster angle increases from 0° to 90° for both level of contour number. The plots of flexural strength and flexural strain exhibit similar trends. The effect of raster angle is much higher when the contour number is at a low level than when it is at its high level. This is due to the fact that the high contour number improves the flexural properties of parts by resisting the applied load more than those parts with a single contour. The flexural modulus decreases drastically when the raster angle increases from 0° to 90° for parts with a single contour, compared to the parts with five contours, as shown in the middle plot of Figure 17.

**Figure 18:**
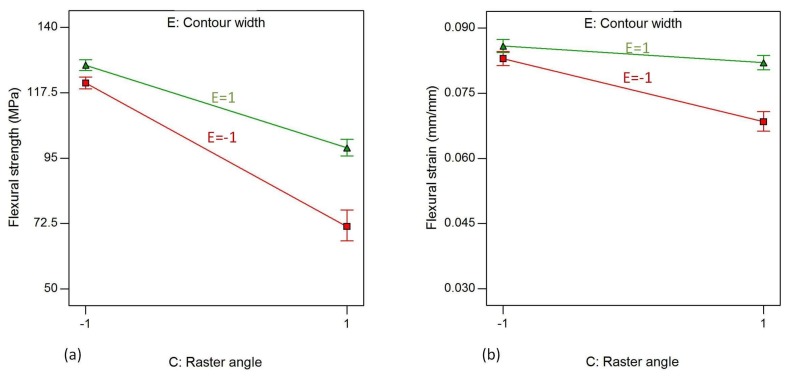
Interaction effect of raster angle and contour width on (**a**) flexural strength and (**b**) flexural strain, while air gap, raster width and contour number are kept at their highest level.

The interaction effects of C (raster angle) and E (contour width) on responses, flexural strength and flexural strain are shown in Figure 18. The other factors are kept at their high level, as previously. The interaction effect has similar trends with the CD effects, as contour number and contour width show a similar effect on both flexural strength and flexural strain.

**Figure 19:**
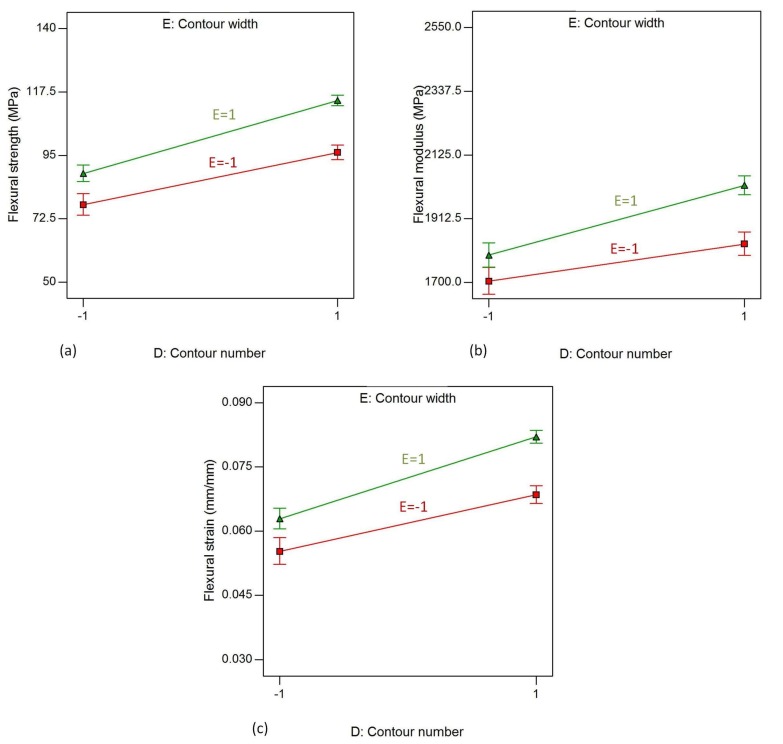
Interaction effect of contour number and contour width on (**a**) flexural strength; (**b**) flexural modulus and (**c**) flexural strain.

The interaction effect of D (contour number) and E (contour width) on responses, flexural strength, flexural modulus and flexural strain are shown in Figure 19. The responses increase when the contour number is increased from one to five for both low and high levels of contour width, which are 0.4064 mm and 0.7814 mm, respectively, while the other factors remain constant. The lowest of all responses are registered for this combination of factor levels: air gap = 1, raster width = 1, raster angle = 1, contour number = −1 and contour width = −1. These responses are 52.89 MPa for flexural strength and 0.03423 for flexural strain.

### 4.7. Optimization of Process Parameters

The FDM process is an additive manufacturing process with many factors that have conflicting advantages for the performance of the produced parts. Hence, it is important to optimize these factors to maximize the flexural properties of the parts. The desirability function is used in this study to determine the optimum process parameters that simultaneously maximize multiple responses. In the numerical optimization process [25], the desired goal for each of the factors and responses is chosen; weights are assigned to each goal, if necessary, to adjust how the optimization process searches for the best solution and, if any of the goals has a different level of importance, it is adjusted by assigning it a relative value. For the current study, the desired goals of the factors are set to be in the range between the lower and higher limits except for the air gap, which is set to the higher limit since the lower limit has drawbacks as discussed in Section 4.1. These desired goals are detailed in Table 4. The goals for the responses are set to maximize them. The weights and importance levels of all the goals are set to be identical, as they are all equally important.

The desirability function optimization approach uses an objective function D(X) given in Equation (8). The objective function reflects the desirable ranges for each response (di) for the number of responses n. The optimization of individual responses is done separately, using the relations shown in Equation (9) and then collected in the combined objective function in Equation (8). The combined objective function (Equation (8)) is a geometric mean of all the regression models developed in this study and discussed at the beginning of Section 4.

(8) $$D={\left(d_{1}\;x\;d_{2}x\ldots x\;d_{n}\right)}^{\frac{1}{n}}={\left(\overset{n}{\underset{i=1}{\prod }}d_{i}\right)}^{\frac{1}{n}}$$

(9) $$d_{i}=0,\;Y_{i}\leq Low_{i}\;d_{i}=\frac{Y_{i}-Low_{i}}{High_{i}-Low_{i}},\;Low_{i}<Y_{i}<High_{i}\;\;d_{i}=1,\;Y_{i}\geq High_{i}$$

where *Y_i_* is the response, and *Low_i_* and *High_i_* represent the lower and higher limits, respectively.

**Figure 20:**
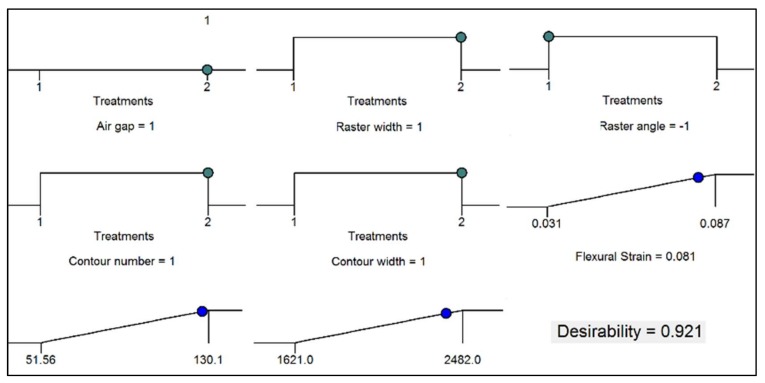
Optimal parameters’ combination with the predicted optimal responses.

**Figure 21:**
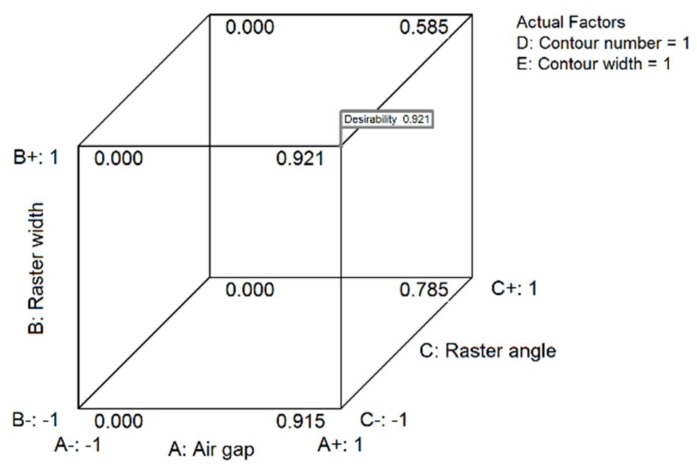
Cube desirability showing values at different parameter combinations.

The plots in Figure 20 illustrate the result of numerical optimization. The figure shows the optimal level of parameter combination that result in an optimal response. The optimal levels of parameters obtained are high-level air gap (0.000 mm), high-level raster width (0.7814 mm), low-level raster angle (0°), high-level contour number (five) and high-level contour width (0.7814 mm). The predicted optimal responses registered for this combination of parameters are 126.996 MPa flexural strength, 2399.803 MPa flexural modulus and 0.081 flexural strain, with desirability of 0.921, as shown in the 3D-cube plot in Figure 21. These predicted optimal responses are moderately in agreement with the experimental result for this combination of parameters.

## 5. Conclusions

The investigation has been carried out to study the effect of different process parameters on the flexural properties of FDM-processed materials. The study was conducted on ULTEM 9085 polymeric material produced by a Fortus 450 mc FDM machine: Although this material type is a high-performance material that has potential application in the aerospace, automotive and military industries, it has not been significantly researched. In the study reported in this article, a full factorial design for the experiment was used to investigate the performance of the material, and the following conclusions can be drawn.

- The FDM process is a complex additive manufacturing technology with different processing parameters of conflicting effects. These parameters have their own advantages and disadvantages in respect of the flexural properties of the material.
- All the considered parameters have importance and effects, especially when the parts are produced with a raster angle other than 0°. When the raster angle is 0°, the effects of the parameters are less visible and less important.
- From the investigation, the effect of the factors can be arranged as most important (raster angle and raster width), important (contour number and contour width) and less important (air gap).
- Maximum responses are registered for the parameter combination of low-level air gap (−0.0254 mm), high-level raster width (0.7814 mm), low-level raster angle (0°), low level of contour number (one) and high level of contour width (0.7814 mm). Moreover, the minimum response is registered for high-level air gap (0.0000 mm), high-level raster width (0.7814 mm), high-level raster angle (90°), low level of contour number (one) and low level of contour width (0.4064 mm).
- Alhough many reports recommend a minus air gap, with some restrictions for ABS material, this study concludes that the disadvantage of the air gap is bigger than its advantage, and its effect can differ between two different materials. Thus, the use of a minus air gap for ULTEM 9085 material is not recommended.
- Raster width and contour width were considered as similar parameters to road width in different studies [4,6,7]. However, from this study it can be concluded that these are two different parameters with completely dissimilar effects and they need to be considered separately.
- The optimal levels of parameters achieved are 0.000 mm air gap, 0.7814 mm raster width, 0° raster angle, contour number of 5, and 0.7814 mm contour width, resulting in flexural strength of 126.996 MPa, flexural modulus of 2399.803 MPa, and 0.081 flexural strain.

## Figures and Tables

**Table 1:** FDM process parameters with their respective levels.

Factors			Levels	
Name	Units	Symbol	Low (−)	High (+)
Air gap	mm	A	−0.0254	0.0000
Raster width	mm	B	0.4064	0.7814
Raster angle	degree	C	0.0000	90.0000
Contour number	-	D	1.0000	5.0000
Contour width	mm	E	0.4064	0.7814

**Table 2:** Full factorial design matrix with measured values of responses.

Run Order	Standard Order	A	B	C	D	E	Flexural Strain at Maximum Flexural Strength (mm/mm)	Flexural Strength (MPa)	Flexural Modulus (MPa)
32	1	−1	−1	−1	−1	−1	0.08351	122.20	2232.5
16	2	1	−1	−1	−1	−1	0.08151	123.55	2351.00
29	3	−1	1	−1	−1	−1	0.08002	126.05	2389.00
13	4	1	1	−1	−1	−1	0.08011	124.65	2385.50
30	5	−1	−1	1	−1	−1	0.06141	100.86	2108.50
14	6	1	−1	1	−1	−1	0.04279	72.87	1991.50
22	7	−1	1	1	−1	−1	0.04198	70.54	1948.50
6	8	1	1	1	−1	−1	0.03423	52.89	1650.00
31	9	−1	−1	−1	1	−1	0.08336	121.75	2232.00
15	10	1	−1	−1	1	−1	0.08094	123.50	2294.50
23	11	−1	1	−1	1	−1	0.08331	124.75	2241.00
7	12	1	1	−1	1	−1	0.07887	121.05	2281.50
24	13	−1	−1	1	1	−1	0.07157	109.35	2146.50
8	14	1	−1	1	1	−1	0.05445	93.54	2098.50
18	15	−1	1	1	1	−1	0.04697	78.27	1946.50
2	16	1	1	1	1	−1	0.04191	70.41	1875.50
28	17	−1	−1	−1	−1	1	0.08430	125.25	2304.50
12	18	1	−1	−1	−1	1	0.07931	125.80	2390.50
25	19	−1	1	−1	−1	1	0.08035	125.95	2429.50
9	20	1	1	−1	−1	1	0.07952	124.85	2372.50
26	21	−1	−1	1	−1	1	0.06292	106.05	2207.00
10	22	1	−1	1	−1	1	0.04448	79.92	2137.50
19	23	−1	1	1	−1	1	0.03825	68.52	1995.00
3	24	1	1	1	−1	1	0.03486	58.19	1690.00
27	25	−1	−1	−1	1	1	0.08146	126.30	2345.50
11	26	1	−1	−1	1	1	0.08036	124.70	2384.50
20	27	−1	1	−1	1	1	0.08094	125.95	2333.50
4	28	1	1	−1	1	1	0.08015	124.50	2389.50
21	29	−1	−1	1	1	1	0.08219	121.45	2306.50
5	30	1	−1	1	1	1	0.07834	116.45	2249.00
17	31	−1	1	1	1	1	0.07571	113.85	2223.50
1	32	1	1	1	1	1	0.07593	104.50	2106.0

**Table 3:** Analysis of variance (ANOVA) table for the regression model developed for the responses.

Source	Sum of Squares	Degree of Freedom	Mean Square	F Value	*p*-Value Prob > F	Remark
**Flexural Strength (1)**						
Block	5.74 × 10^10^	1	5.74 × 10^10^	-	-	-
Model	2.33 × 10^12^	10	2.33 × 10^11^	115.05	<0.0001	significant
A-A	5.21 × 10^10^	1	5.21 × 10^10^	25.68	<0.0001	significant
B-B	8.73 × 10^10^	1	8.73 × 10^10^	43.06	<0.0001	significant
C-C	1.63 × 10^12^	1	1.63 × 10^12^	803.47	<0.0001	significant
D-D	1.13 × 10^11^	1	1.13 × 10^11^	55.74	<0.0001	significant
E-E	8.60 × 10^10^	1	8.60 × 10^10^	42.44	<0.0001	significant
AC	3.69 × 10^10^	1	3.69 × 10^10^	18.18	<0.0001	significant
BC	1.04 × 10^11^	1	1.04 × 10^11^	51.41	<0.0001	significant
CD	1.40 × 10^11^	1	1.40 × 10^11^	69.26	<0.0001	significant
CE	3.79 × 10^10^	1	3.79 × 10^10^	18.71	<0.0001	significant
DE	4.56 × 10^10^	1	4.56 × 10^10^	22.5	<0.0001	significant
Residual	1.05 × 10^11^	52	2.03 × 10^09^	-	-	-
Cor Total	2.50 × 10^12^	63	-	-	-	-
**Flexural Modulus (2)**						
Block	1.97 × 10^19^	1	1.97 × 10^19^	-	-	-
Model	4.24 × 10^20^	10	4.24 × 10^19^	47.96	<0.0001	significant
A-A	3.27 × 10^18^	1	3.27 × 10^18^	3.69	0.0602	insignificant
B-B	1.82 × 10^19^	1	1.82 × 10^19^	20.62	<0.0001	significant
C-C	2.61 × 10^20^	1	2.61 × 10^20^	294.53	<0.0001	significant
D-D	4.74 × 10^18^	1	4.74 × 10^18^	5.36	0.0246	significant
E-E	3.49 × 10^19^	1	3.49 × 10^19^	39.5	<0.0001	significant
AB	5.03 × 10^18^	1	5.03 × 10^18^	5.69	0.0207	significant
AC	2.01 × 10^19^	1	2.01 × 10^19^	22.77	<0.0001	significant
BC	4.295 × 10^19^	1	4.295 × 10^19^	48.55	<0.0001	significant
CD	2.682 × 10^19^	1	2.682 × 10^19^	30.32	<0.0001	significant
DE	7.58 × 10^18^	1	7.58 × 10^18^	8.57	0.0051	significant
Residual	4.60 × 10^19^	52	8.85 × 10^17^	-	-	-
Cor Total	4.90 × 10^20^	63				
**Flexural Strain (3)**						
Block	1.75 × 10^−13^	1	1.75 × 10^−13^	-	-	-
Model	7.41 × 10^−6^	10	7.41 × 10^−7^	64.51	<0.0001	significant
A-A	1.90 × 10^−7^	1	1.90 × 10^−7^	16.51	0.0002	significant
B-B	2.68 × 10^−7^	1	2.68 × 10^−7^	23.33	<0.0001	significant
C-C	4.79 × 10^−6^	1	4.79 × 10^−6^	417.29	<0.0001	significant
D-D	6.039 × 10^−7^	1	6.039 × 10^−7^	52.56	<0.0001	significant
E-E	2.278 × 10^−7^	1	2.278 × 10^−7^	19.83	<0.0001	significant
AB	7.14 × 10^−8^	1	7.14 × 10^−8^	6.22	0.0159	significant
BC	1.097 × 10^−7^	1	1.097 × 10^−7^	9.55	0.0032	significant
CD	5.821 × 10^−7^	1	5.821 × 10^−7^	50.67	<0.0001	significant
C E	3.23 × 10^−7^	1	3.23 × 10^−7^	28.08	<0.0001	significant
DE	2.42 × 10^−7^	1	2.42 × 10^−7^	21.07	<0.0001	significant
Residual	5.97 × 10^−7^	52	1.15 × 10^−8^	-	-	-
Cor Total	8.01 × 10^−6^	63	-	-	-	-
**(1)** R^2^ = 0.9568, Adjusted R^2^ = 0.9484, Predicted R^2^ = 0.9345, Adequate Precision = 34.576						
**(2)** R^2^ = 0.9022, Adjusted R^2^ = 0.8834, Predicted R^2^ = 0.8518, Adequate Precision = 24.531						
**(3)** R^2^ = 0.9254, Adjusted R^2^ = 0.9111, Predicted R^2^ = 0.8870, Adequate Precision = 24.099						

**Table 4:** Constraints of factors and responses used for the optimization.

Name	Goal	Lower Limit	Upper Limit	Lower Weight	Higher Weight	Importance
Air gap	Is equal to 1	−1	+1	1	1	3
Raster width	In range	−1	+1	1	1	3
Raster angle	In range	−1	+1	1	1	3
Contour number	In range	−1	+1	1	1	3
Contour width	In range	−1	+1	1	1	3
Flexural strength (MPa)	Maximize	51.56	130.10	1	1	3
Flexural modulus (MPa)	Maximize	1621	2482	1	1	3
Flexural strain	Maximize	0.03116	0.08682	1	1	3
